# Influence of glucose and insulin in human adipogenic differentiation models with adipose-derived stem cells.

**DOI:** 10.1080/21623945.2019.1636626

**Published:** 2019-07-06

**Authors:** Michaela Kolodziej, Sarah Strauss, Andrea Lazaridis, Vesna Bucan, Jörn W. Kuhbier, Peter M. Vogt, Sören Könneker

**Affiliations:** Department of Plastic, Aesthetic, Hand and Reconstructive Surgery, Hannover Medical School, Hanover, Germany

**Keywords:** Adipogenic differentiation, fat cell augmentation, glucose, human adipose-derived stem cells (hASC), insulin, lipid accumulation

## Abstract

Autologous fat grafting represents an attractive source for tissue engineering applications in the field of reconstructive medicine. However, in adipogenic differentiation protocols for human adipose-derived stem cells, the concentration of glucose and insulin varies considerably. With the intent to gain maximum tissue augmentation, we focused on the late phase of adipogenesis. In this study, we modified the differentiation protocol for adipose-derived stem cells by prolongation of the induction period and the application highly concentrated glucose and insulin. Human adipose-derived stem cells were isolated from subcutaneous depots and differentiated in a standard induction medium for the first two weeks, followed by two weeks with varying glucose and insulin concentrations. Morphological changes assessed using Oil-Red-O staining were examined for corresponding alterations in the expression of the adipogenic markers peroxisome proliferator-activated receptor gamma (PPARγ) and lipoprotein lipase (LPL). Furthermore, glucose and lactate levels in conditioned media were monitored over the period of differentiation. We found high-glucose media increasing the level of lipid accumulation and the size of single droplets whereas insulin significantly showed a dose-dependent negative effect on fat storage. However, whereas high glucose stimulated PPARγ transcription, expression levels in insulin-treated cells remained constant. Results permit assumptions that a high-glucose medium intensifies the degree of differentiation in mature adipocytes providing conditions to promote graft volume while we have identified highly concentrated insulin treatment as an inhibitor of lipid storage in the late adipogenic differentiation.

## Introduction

Soft tissue engineering is a growing field that addresses the clinical challenges associated with the structural and functional therapy of defects caused by congenital deformities, post-operative or post-traumatic loss of soft tissue. Nevertheless, the clinical application of fat tissue grafting is often limited by an inconsistent availability and surgeons are still faced with challenging the resulting loss of donor substance [1]. Thus, patients would greatly benefit from future grafting methods applying *ex vivo* augmentation of minimal autologous fat removal as a source for reconstructive therapies.

Since their isolation of the stromal vascular fraction and characterisation was first established in 2001, there was a growing interest in adipose-derived stem cells (ASC) as a source for regenerative medicine applications [2]. Especially, their accessibility from subcutaneous depots with minimal morbidity and ability of multilineage differentiation, including the adipogenic lineage, offer a great potential for autologous reconstruction therapies of soft tissue defects [3]. The use of autologous stem cells has been suggested as a promising technique avoiding the disadvantages of foreign materials like undesired immune responses and the risk of infections [4]. In this case, subcutaneous fat tissue has not only been shown to be the richest source for the isolation of mesenchymal stem cells, it is also thought to have the highest differentiation potential [5]. The possibility to store excess metabolic energy in form of triglycerides is a characteristic trait of fat tissue that results from hyperplasia, the proliferation of undifferentiated precursor cells and hypertrophy, the increase in individual cell size in mature adipocytes. Latter can either be achieved through the direct uptake of plasma-free-fatty acids or glucose. In this regard, insulin acts as a fundamental element in the energy homeostasis regulating glucose metabolism and lipogenesis [6]. Although research has been widely carried out on how adipogenic stimulants affect the induction period of the differentiation process, so far, the effect on late adipogenic differentiation has not been completely investigated. Indeed, glucose and insulin are known to be essential ingredients of the differentiation media; however, in literature, there is still no consensus about their optimal application. Differentiation protocols showed varying concentrations from 5.5 mM to 25 mM for glucose and from 0.1 µM to 10 µM for insulin application (Table 1).

**Table 1.** Comparative assessment of adipogenic media supplementation for the *in vitro* differentiation of adipose-derived stem cells (ASC). Reference for standard differentiation media established in our laboratory is underlined.

Source	Basal Media	Glucose (mM)	Insulin (µM)	Further Supplementation
[30]	DMEM/F12	17.5	1.7	10% FCS, 0.5 mM IBMX, 1 µM dexamethasone, 0.1 mM indomethacin
[31]	DMEM/F12	17.5	0.86	10% FCS, 0.5 mM IBMX, 1 M dexamethasone, 1 M rosiglitazone
[32]	DMEM/F12	17.5	0.5	0.25 mM IBMX, 0.1 µM dexamethasone, 33 μM biotin, 1 μM troglitazone, 10 μg/ml transferrin, 200pM T3
[33]	DMEM/F12	17.5	0.1	0.25 IBMX, 1 µM dexamethasone,
[34]	MEM	5.5	10	10% FCS, 0.5 mM IBMX, 1 µM dexamethasone, and 0.2 mM indomethacin, 1% antibiotic/antimycotic
[35]	DMEM/F12	17.5	0.17	10% FCS, 0.45 mM IBMX, 0.1 µm dexamethasone, 0.2 mM indomethacin, 1 µm rosiglitazone
[36]	High glucose DMEM	25	1.7	10% FCS, 0.5 IBMX, 0.2 mM indomethacin
[37]	DMEM/F12	17.5	0.85	0.1 M IBMX, 1 μM dexamethasone, 100 μM ascorbic acid, 20 nM sodium selenite, 0.2 nM triiodothyronine, 1 μM rosiglitazone

With the intent to address this issue, we created an *in vitro* model of monolayer cultures to examine the effect of glucose and insulin on lipid accumulation, gene expression, metabolism and viability of adipogenic differentiating ASC. In this context, we aimed to enhance human fat cell augmentation during the late adipogenic differentiation.

## Material and methods

### ASC isolation

Subcutaneous abdominal adipose tissue was obtained from two male and four female patients aged between 25 and 41 years (mean 34 years) with a mean body mass index (kg/m^2^) ± standard deviation of 26 ± 2.1 undergoing elective abdominal reduction surgery at the Hannover Medical School, Department of Plastic, Aesthetic, Hand and Reconstructive Surgery. All patients provided their written informed consent according to the approval procedures by the local ethics committee at the Hannover Medical School (reference no. 3073–2016). Referring to preoperative examination the patients were free from diabetes and other severe metabolic diseases. The procedure followed previously described standard protocols for mesenchymal stem cell isolation with slight modifications [7,8]. Briefly, the tissue obtained was separated from the skin and minced to a fine consistency with scissors. The extracellular matrix was enzymatically digested with 0.2% (w/v) collagenase of Type I (Biochrom AG, C1-22; Berlin, Germany) in Hank’s Balanced Salt Solution (Sigma-Aldrich, Munich, Germany) under gentle agitation overnight at 4°C and the following day at 37°C for 1 h. The digested specimen was then centrifuged at 300xg for 5 min to segregate the stromal vascular fraction from the floating mature adipocytes. After removing the aqueous intermediate layer, Hank’s with 5% bovine serum albumin (BSA; Sigma-Aldrich, A9418; Munich, Germany) was added, followed by centrifugation for 10 min. After repeating this washing step, supernatant was discarded and cell pellets were resuspended in ASC growth media (GM) comprised of DMEM/F12 (Biochrom, FG 4815; Berlin, Germany), 10% fetal calf serum (FCS; Biochrom, S0615; Berlin, Germany), 50 U/mL penicillin/streptomycin (Biochrom, A2212; Berlin, Germany) and 0.173 mM ascorbate-2-phosphate (Sigma-Aldrich, A8960; Munich, Germany) and filtered through a 100 µm nylon mesh to remove cellular debris. Freshly isolated cells of the stromal vascular fraction were directly used for cultivation and following investigations.

### ASC characterization

Cell surface phenotype of freshly isolated human ASC at passage 0 (P0) was analysed by flow cytometry. Achieving confluency after average time of 2 weeks in 10% FCS containing GM, cells were detached with 0.2% (v/v) EDTA/PBS (Thermo Fisher, AM9260G; Waltham, USA), passed through a 100 µm nylon sieve and Trypan Blue Dye Exclusion (Countess Automated Cell Counter, Invitrogen; Carlsbad, USA) was used for assessing the cellular viability. Samples were blocked with 1% (w/v) BSA/PBS and permeabilized with 0,1% (v/v) Triton X-100/PBS (Sigma-Aldrich, X100; Munich, Germany) for 30 min at 4°C. Conjugated mouse monoclonal antibodies were used according to the manufacturer’s recommendations, followed by incubation for 1 h at room temperature in the dark. The antibodies used were: CD11b-PE-Cy7, CD13-PE, CD19-FITC, CD31-FITC, CD34-PC7, CD44-PE, CD45-PC5, CD73-PE, CD90-PC5, CD105-PE and HLA-DR-FITC [9,10]. Manufacturer and catalogue numbers of purchased antibodies are listed in Table 2. Anti-human isotype-matched antibodies and cells without antibodies served as controls. Fluorescence-activated cell sorting of stained cells was performed using a flow cytometer (Beckman Coulter FC500; Brea, USA) equipped with a CXP software for data analysis.

**Table 2.** Phenotypic characterization of isolated human adipose-derived stem cells (hASC) at passage 0. Columns show manufacturers with catalogue numbers of used antibodies, donors (1–6) and mean ± SEM. Data are presented as percentage of positive cells for the particular surface marker measured by flow cytometry of 5000 cells.

Antigen	Manufacturer	1	2	3	4	5	6	mean ± SEM
CD 11b	eBioscience; 25–0118	2	6	15	7	2	5	6 ± 2
CD 13	Biolegend; 301,703	95	93	89	93	79	87	89 ± 2.4
CD 19	Beckmann Coulter; A07768	1	0	4	11	1	2	3 ± 1.7
CD 31	Beckmann Coulter; IM1431U	0	2	9	6	0	11	5 ± 1.9
CD 34	Beckmann Coulter; A21691	0	32	11	8	6	11	11 ± 4.5
CD 44	Biolegend; 338808	96	98	97	98	81	99	95 ± 2.8
CD 45	Beckmann Coulter; IM2652U	0	10	21	11	12	46	17 ± 6.5
CD 73	Biolegend; 344003	91	90	68	93	90	56	81 ± 6.3
CD 90	Beckmann Coulter; PN IM3703	96	96	88	97	99	69	91 ± 4.6
CD 105	Beckmann Coulter; PN A07414	17	93	75	67	33	79	61 ± 12
HLA-DR	Imgenex; IMG-6881AF-488	2	2	13	8	0	31	9 ± 4.8

### Cell differentiation

**Figure 1.**
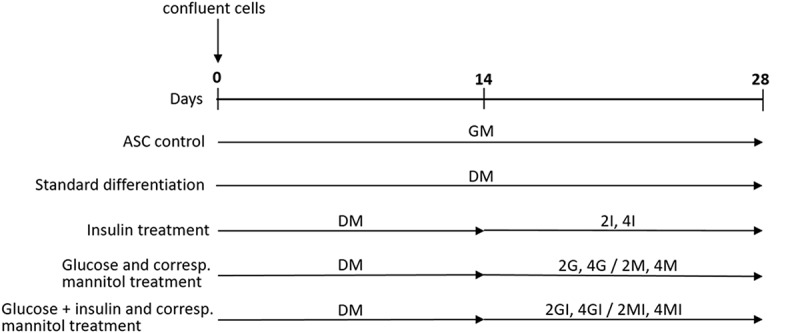
Schematic timeline of the adipogenic differentiation protocol. Human adipose-derived stem cells (hASC) were grown to confluence (defined as day 0) and differentiated in standard media (DM) or with glucose and insulin for a period of 28 days. Cells cultured in growth media (GM) for equal time served as an undifferentiated control and D-Mannitol was consistently used as an osmotic control for glucose treated cells.

Cells which were seeded directly after isolation and expanded to reach confluence, defined as P0, were directly cultivated in a 37°C humidified atmosphere containing 5% CO2 and media was exchanged twice per week. After achieving 100% confluence, adipogenic differentiation was initiated using a standard differentiation media (DM) which is established in our laboratory, composed of GM with 1 µM dexamethasone (Sigma-Aldrich, D1756; Munich, Germany), 0.5 mM 3-isobutyl-1-methylxanthine (IBMX, Sigma-Aldrich, I5879, Munich, Germany), 0.1 mM indomethacin (Sigma-Aldrich, I7378; Munich, Germany) and 1.7 µM human insulin (Biochrom, W1493950288; Berlin, Germany). After 14 days, cells were either kept in DM (17.5 mM glucose, 1.7 µM Insulin) for further 14 days, in DM with a twofold or fourfold concentration of glucose and insulin (2GI: 35 mM glucose, 3.4 µM insulin; 4GI: 70 mM glucose, 6.8 µM insulin) or the twofold or fourfold concentration of glucose (2G, 4G) or insulin (2I, 4I) alone. Cells cultured in GM served as an undifferentiated control and D-Mannitol was used in all experiments as an osmotic control for glucose-treated cells (2MI, 4MI, 2M, 4M). (Figure 1, Table 3)

**Table 3.** Supplementation of glucose, insulin and mannitol used in cell culture media with reference to the standard differentiation medium for the adipogenic differentiation of human adipose-derived stem cells (hASC).

Medium	Glucose (mM)	Insulin (µM)	Mannitol (mM)
GM: Growth medium	17.5		
DM: Standard differentiation medium	17.5	1.7	
2I: 2-fold insulin concentration	17.5	3.4	
4I: 4-fold insulin concentration	17.5	6.8	
2G: 2-fold glucose concentration	35	1.7	
2M: Mannitol control for 2G	17.5	1.7	17.5
4G: 4-fold glucose concentration	70	1.7	
4M: Mannitol control for 4G	17.5	1.7	52.5
2GI: 2-fold glucose and insulin concentration	35	3.4	
2MI: Mannitol control for 2GI	17.5	3.4	17.5
4GI: 4-fold glucose and insulin concentration	70	6.8	
4MI: Mannitol control for 4GI	17.5	6.8	52.5

### Cell viability analysis

To assess the effect of the media on the viability of cultured cells, a live/dead assay (Invitrogen, L3224; Carlsbad, USA) was performed according to the manual. ASC were seeded on cover glasses, grown to confluence and cultivated with the particular media for 28 days. After washing the cells with PBS, samples were incubated with live/dead solution consisting of ethidium-homodimer-1 and calcein AM. Simultaneous illustration of live and dead cells was performed using a fluorescence microscope (Biozero BZ-8000, Keyence, Osaka, Japan) and analysed qualitatively.

### Oil-Red-O staining

For the imaging of lipid accumulation, cells were stimulated in six-well culture plates with each medium for 28 days and stained with Oil-Red-O (Serva, Heidelberg Germany) as described previously [11]. Prior to staining, cultures were gently rinsed with PBS and fixed with 4% (w/v) paraformaldehyde for 30 min. Oil-Red-O stock solution (0.5% (w/v) in isopropanol) was diluted 3:2 with distilled water and filtered through a 0.22 µm filter. Cells were covered with working solution for 10 min and after a brief treatment in 60% (v/v) isopropanol, counterstaining with haematoxylin (Merck, 1376; Darmstadt, Germany) for 5 min followed. Stain was visualized by light microscopy (Olympus CK40, Tokyo, Japan) and images were obtained randomly from 10 representative fields with a digital camera (Digit Micro camera Olympus Color View II, Tokyo, Japan) at 20x magnification. As described previously, neutral lipid vacuoles were then quantified using the java-based image processing program ImageJ [12]. After obtaining images, the intracellular dye was leached from the stained culture plates with isopropanol and its absorbance was analysed by spectrophotometry (NanoDrop-1000 Spectrophotometer, PEQLAB; Erlangen, Germany) at 518 nm.

### Quantitative real-time PCR

Previously, our laboratory had identified RPS12 and RPL37 as ribosomal genes with sufficient stability addressing the lack of suitable reference genes for hASC before and after three-lineage differentiation. Genes with the smallest mean value of the standard deviation were selected tissue specifically for the adipogenic lineage. To validate gene stability for our experiment, two-step reverse transcription-polymerase chain reaction (qRT-PCR) was performed in three replicas for all control and treatment groups (n = 6). Quality of selected primer pairs was validated by analysing melting curves and verifying product size on 4% ethidium bromide containing agarose gel (Biozym, 840004; Oldendorf, Germany).

Total RNA was isolated from cells after 4 weeks of adipogenic differentiation using the Macherey-Nagel NucleoSpin kit (Macherey Nagel, 740955.50; Düren, Germany) following the manufacturer’s protocol. RNA samples were tested for purity, quantified by absorbance at 260 nm (NanoDrop-1000 Spectrophotometer, PEQLAB; Erlangen, Germany) and stored at −80°C until amplification. Extracted RNA was highly pure (absorption ratio 260/280 > 1,8; 260/230 > 2,0). Integrity of the isolated RNA was verified by gel electrophoresis. Reverse transcription was performed with 1 µg of total RNA using iScript cDNA Synthesis Kit (Bio-Rad; 170–8891; Hercules, USA). The resulting cDNA was diluted to 1:50 and used in a 15 µL PCR mixture containing forward and reserve primers, Sso Fast Evergreen Supermix (Bio Rad, 172–5202; Hercules, USA) and HPLC water. Primer3 software was used to select gene-specific primer sequences and design primers for LPL, PPAR-gamma and the housekeeping genes RPL37 and RPS12 (Table 4). Reactions were performed in triplicate assays using a Thermocycler Bio-Rad iCycler (Hercules, USA). qPCR cycling conditions were as follows: 10 min at 95°C, 30 min at 60°C, 15 min at 70°C and 4°C as holding temperature. Housekeeping gene stability was validated and results were geometrically averaged. Expression of adipogenic markers was relatively quantified by ΔΔCT method [13] and results were normalized to housekeeping genes. Representative PCR products were visualized by electrophoresis on 4% ethidium bromide-stained agarose gel to verify the amplified product size and primer specificity.

**Table 4.** List of reference (RPL37, RPS12) and target (LPL, PPARγ) genes used for qRT-PCR. Forward (F) and reverse (R) sequences of primer pairs and expected amplicon length in base pairs (bp).

Gene	Acc Number^1^	Sequence 5ʹ-> 3’	Product size (bp)
RPL37	NM_000997.4	F: GCCAAGCGCAAGAGAAAGTA (20) R: CACGGAATCCATGCCTGAATC (21)	118
RPS12	NM_001016.3	F: TGACAACAAGAAACTAGGAGAATG (24) R: TACTACACAACTGCAACCAACCAC (24)	91
LPL	NM_000237.2	F: AGCGCTCCATTCATCTCTTCA (21) R: GATTGTTGCAGCGGTTCTTTC (21)	132
PPARγ	NM_015869.4	F: GCTGTTATGGGTGAAACTCTG (21) R: GCGATCTCTGTGTCAACCAT (20)	116

^1^National Center for Biotechnology Information (NCBI)

### Metabolite turnover

During the cells’ exposure to different insulin and glucose concentrations, the conditioned media were collected, pooled weekly and frozen at −20°C for subsequent quantitative analysis with a clinical benchtop blood gas analyser (ABL 800 FLEX, Radiometer Medical ApS; Brønshøj, Denmark). Metabolite turnover was examined by the amperometric measurement of glucose and lactate concentrations in media before and after cultivation.

### Statistical analysis

Experimental data were expressed as mean ± standard error of the mean (SEM). Data were exported to Microsoft Excel® (Microsoft Corporation; Redmond USA) and GraphPad Prism 6 (GraphPad Software; La Jolla, USA) for following calculations and parametric tests. Paired two-tailed t-test was used to assess differences between treatment and control groups with statistical significance determined at p values * <0.05 and ** <0.01.

## Results

### Cells exhibit ASC-typical phenotype characteristics

In culture, hASC adhered to plastic, showed a uniform spindle-like shape and proliferated to confluence within an average time of two weeks. Isolated nonadherent hematopoietic cells and erythrocytes were found, which were removed by media change. To determine the purity of the expanded ASC, isolated from subcutaneous adipose tissue depots, classification of the cell population was performed prior to differentiation using fluorescence activated cell sorting (FACS) for detecting surface markers. Antigen expression is shown in percentage of positive cells for the particular surface marker measured by flow cytometry of 5000 cells (Table 2). P0 cells strongly expressed the characteristic markers for ASC: CD13 (89% ± 2.4%), CD44 (95% ± 2.8%), CD73 (81% ± 6.3%), CD90 (91% ± 4.6%) and CD105 (61% ± 12%) and lack the expression of CD11b (6% ± 2%), CD19 (3% ± 1.7%), CD31 (5% ± 1.9%), CD34 (11% ± 4.5%), CD45 (17% ± 6.5%) and HLA-DR (9% ± 4.8%). However, between different donors, cells showed a considerable inhomogeneity in the expression of CD105 and hematopoietic markers CD34 and CD45.

### Glucose and insulin have no effect on viability

**Figure 2.**
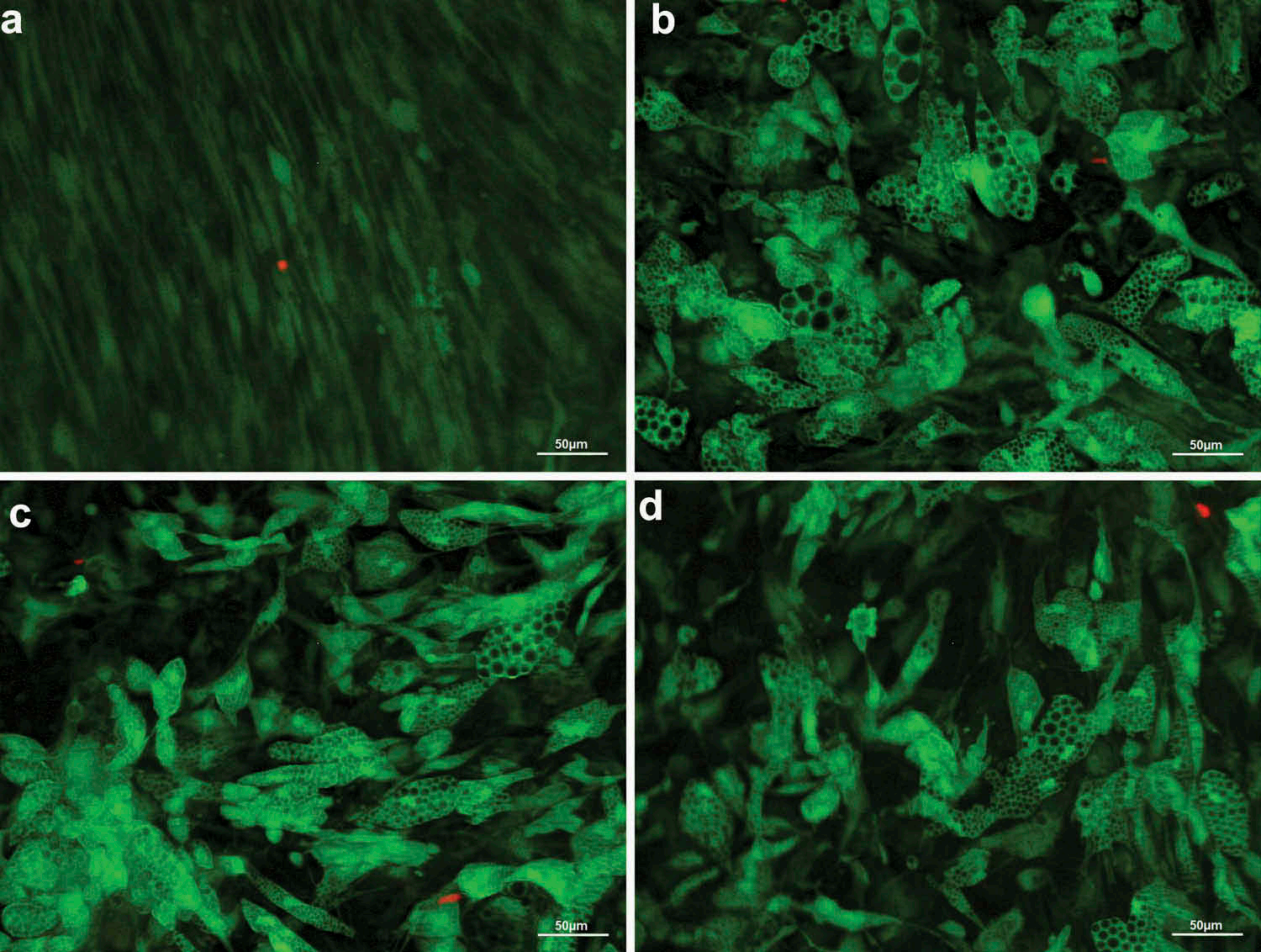
Fluorescence microscopic visualization of live/dead assay after 28 days of adipogenic differentiation of human adipose-derived stem cells. Vital cells with numerous large lipid vacuoles stained green, dead cells stained read. Overlay at 20x magnification, scale bar: 50 µm. (a) undifferentiated control (GM); (b) standard differentiation media (DM); (c) fourfold glucose concentration (4G); (d) fourfold insulin concentration (4I).

High glucose and insulin concentration did not show any effect on cell viability compared to standard differentiation protocol. Throughout culture, no evidence of cell toxicity was found in experimental treated groups. Nearly all cells showed a green fluorescence, suggesting cell viability was unaffected by highly concentrated glucose and insulin. A few dead cells, indicated by red fluorescence, were observed in treatment as well as in control groups (Figure 2).

### Effect of glucose and insulin on lipid accumulation

**Figure 3.**
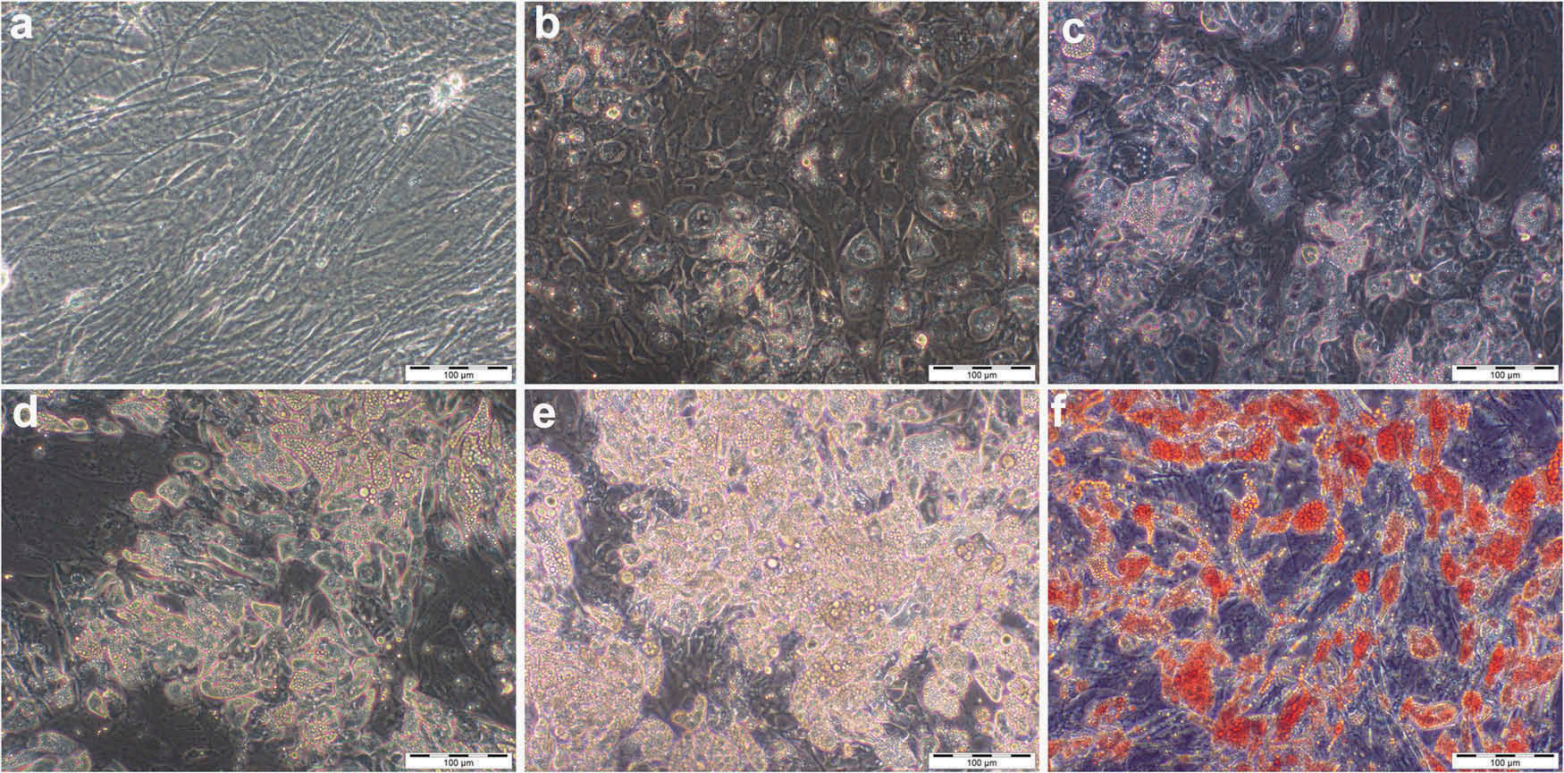
Accumulation of lipid droplets over the time of adipogenic differentiation in DM visualized by light microscopy (20x magnification, scale bar: 100 µm). (a) undifferentiated adipose-derived stem cells (hASC) 7 days after isolation; (b–e) 7, 14, 21 and 28 days after differentiation was induced; (f) Oil-Red-O stained cells, 28 days after differentiation was induced.

**Figure 4.**
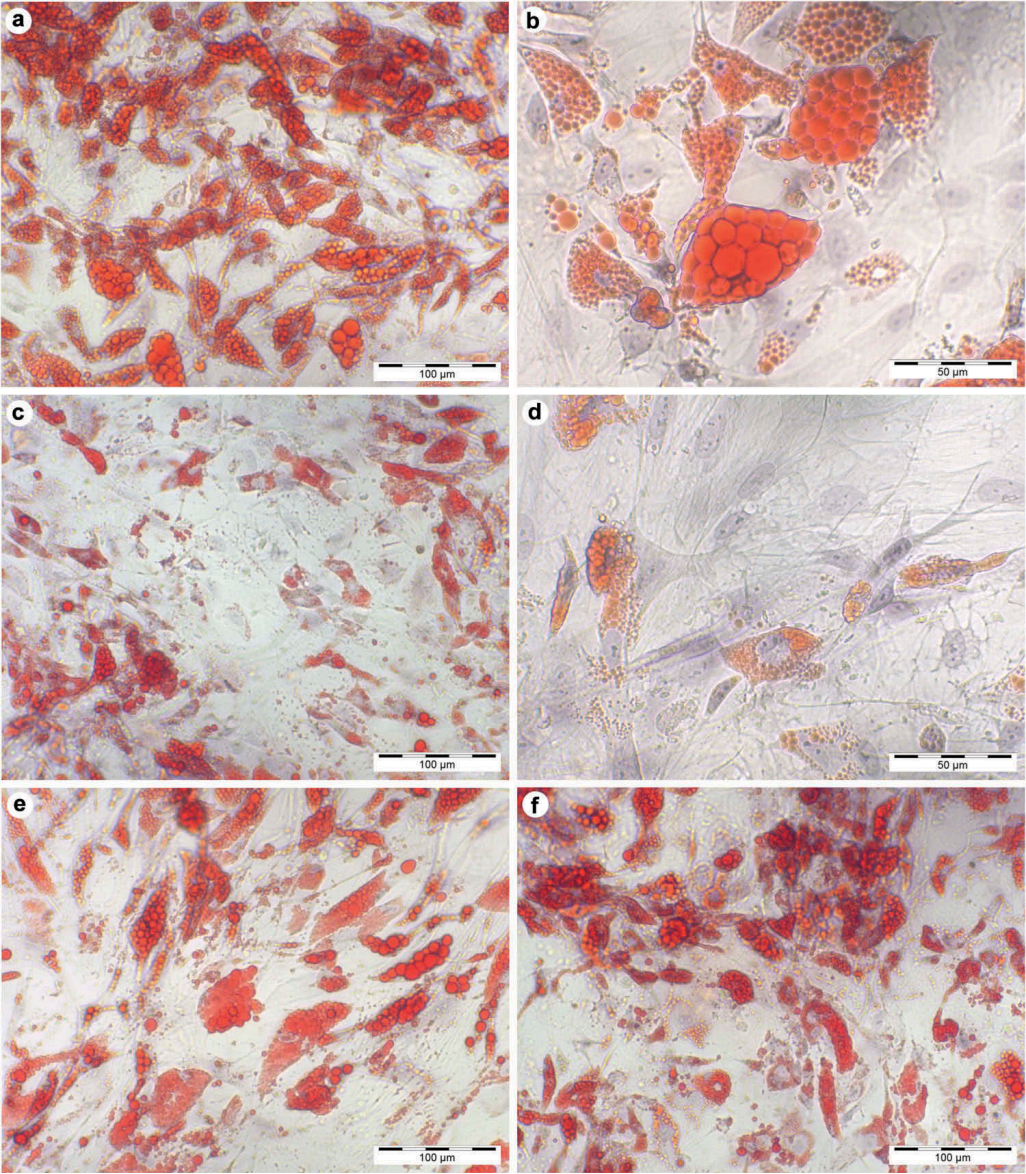
Histological stain after 28 days of differentiation in supplemented adipogenic media. Cultures with fourfold glucose (4G: a, b) and fourfold insulin (4I: c, d) concentrations at 20x (a, c; scale bar: 100 µm) and 40x magnification (b, d; scale bar: 50 µm). Standard differentiation (e) and mannitol treatment as an osmotic control for 4G (f) are presented at 20x magnification. Lipid-containing vacuoles were visualized by Oil-Red-O staining (red), cell nuclei were stained by haematoxylin (blue). Compared to high insulin treated cells, Oil-Red-O stained area was significant higher in fourfold glucose conditions with larger lipid droplets (b) which is characteristic for mature adipocytes.

**Figure 5.**
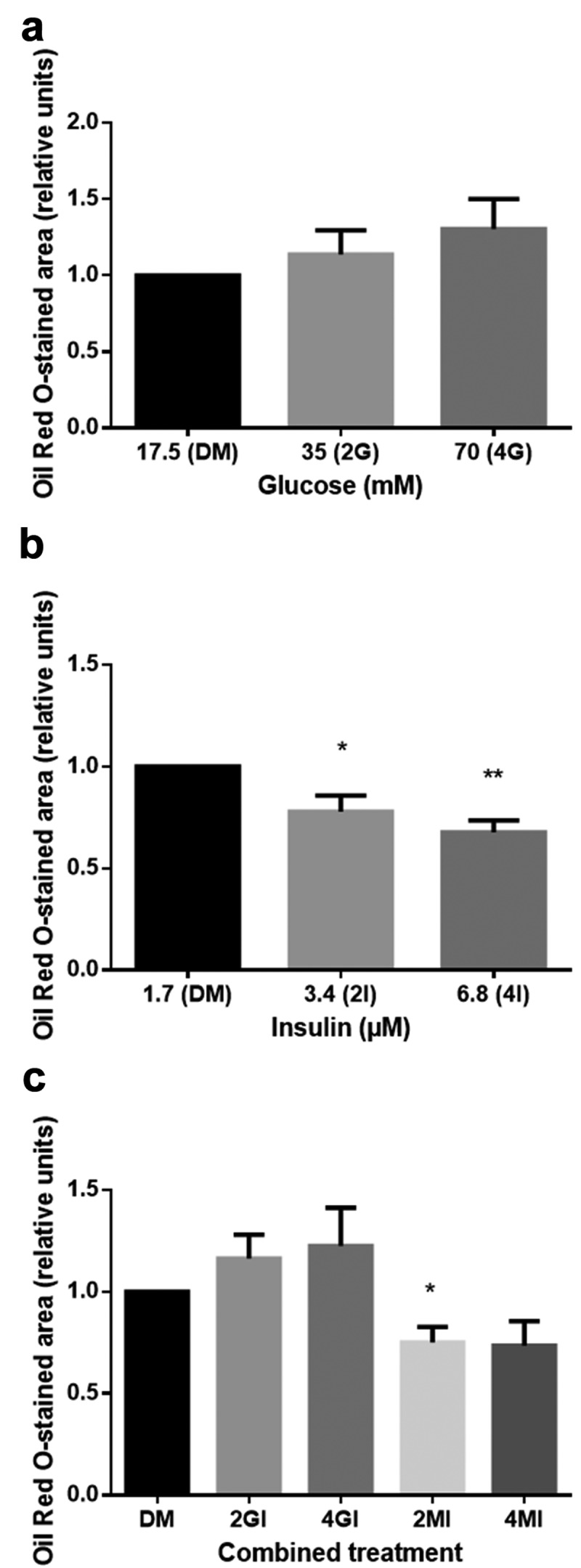
Relative differences in lipid accumulation after 28 days of adipogenic differentiation compared to standard media (DM). Oil-Red-O stained area was quantified in 10 randomly selected microscopic fields by the java-based image processing program ImageJ. Values are presented as mean ± SEM, students t-test was used for statistical analysis; *p < 0.05 and **p < 0.01 for significant results compared to DM. (a) Glucose increases lipid levels, however not significantly (DM: standard differentiation media; 2G, 4G: twofold and fourfold glucose concentration). (b) Insulin significantly reduces lipid accumulation (DM: standard differentiation media; 2I, 4I: twofold and fourfold insulin concentration) (c) which can be partially rescued by glucose (DM: standard differentiation media; 2GI, 4GI: twofold and fourfold glucose and insulin concentration; 2MI, 4MI: mannitol control for combined treatment).

After differentiation was induced, ASC lost their fibroblastic morphology (Figure 3(a)) and adopted a more spherical shape with multiple small lipid droplets which coalesced gradually during culture (Figure 3(b–e)). Cells that were cultured in GM for equal time did not show intracellular lipid accumulation or changes in cell morphology. Intracytoplasmic lipid accumulation was evaluated by light microscopy of Oil-Red-O staining (Figure 3(f)). As shown in Figure 4(a,b), glucose-stimulated cells exhibit a dose-dependent increase both in the number and size of lipid-containing vacuoles. Quantification of the stained area is demonstrated as relative differences compared to DM with the value 1.0 (Figure 5). Cells treated with fourfold glucose concentrations showed a higher percentage of Oil-Red-O stained area; however, these results were not significant. (4G: 1.3 ± 0.2, p > 0.05; Figure 5(a)). In contrast, insulin significantly decreased the level of lipid droplet formation in a dose-dependent manner (2I: 0.78 ± 0.08, p < 0.05; 4I: 0.68 ± 0.06, p < 0.01; Figure 5(b)). Furthermore, supplementing the media with increasing insulin concentrations, the proportion of unilocular lipid droplets was found to be diminished leading to a more immature phenotype with multiple small vacuoles (Figure 4(c,d)). Interestingly, high concentrations of insulin also showed to reduce the stimulatory effect of glucose (4GI: 1.22 ± 0.19; Figure 5(c)). It was not possible to reproduce the effect of glucose by an osmotic equivalent mannitol treatment (Figure 4(f)). Compared to control samples, stained area was even significantly reduced in mannitol + insulin-treated cells (2MI: 0.75 ± 0.08, p < 0.05; 4MI: 0.73 ± 0.12; Figure 5(c)). Results that were obtained by quantitative analysis of light microscopy were confirmed by spectrophotometrically measurement of leached dye.

### Changes in gene expression

**Figure 6.**
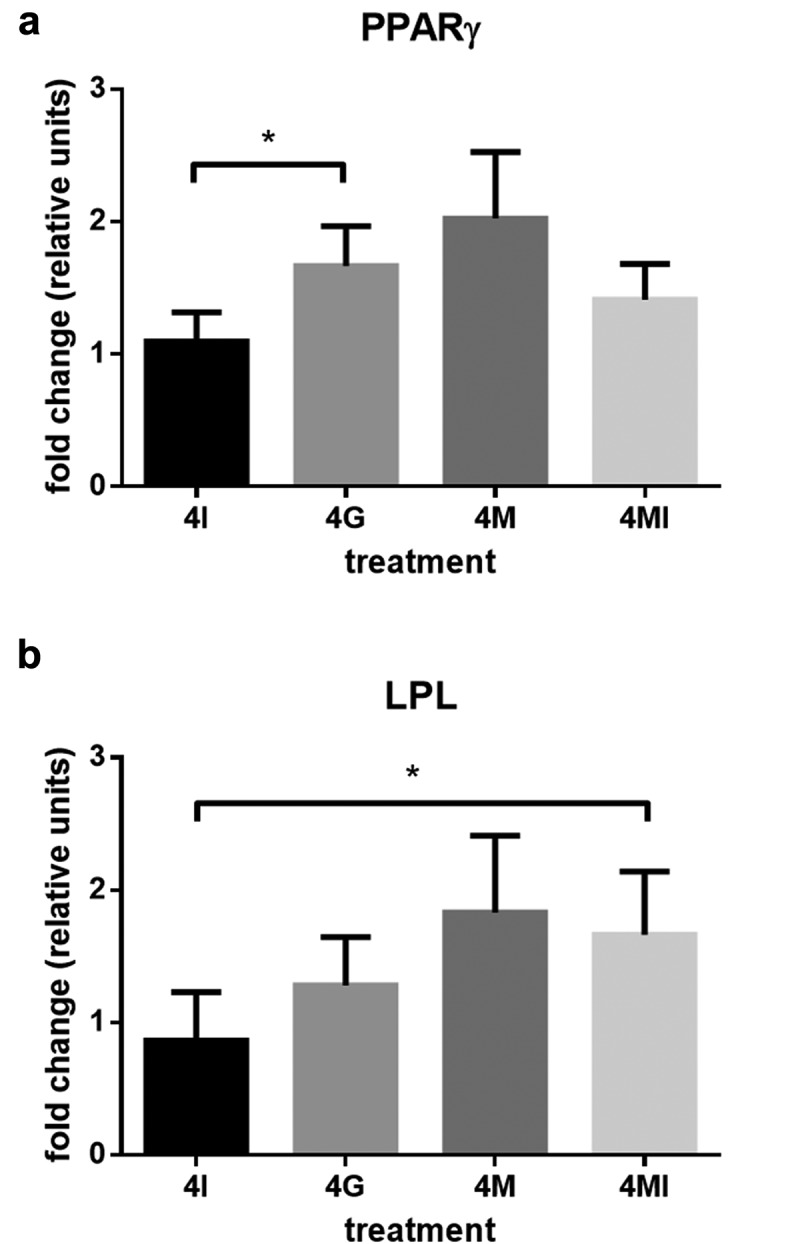
Fold change of PPARγ (a) and LPL (b) gene expression, 28 days after adipogenic differentiation induction, analysed by qRT-PCR. ΔΔCT method was used for relative quantification with RPL37 and RPS12 as reference genes compared to gene expression in standard differentiation media (DM). Mean values are expressed in relative units ± SEM. Statistical comparison between groups was performed with students t-test; *p < 0.05.

To assess whether the observed differences were accompanied by changes in gene expression, the expression of adipogenic markers was analysed by qRT-PCR. The potential reference genes showed a stable expression both in ASC and adipocytes. Intra-assay coefficient of variability in replicas for crossing points was <1%. Although there were no statistically significant results for gene expression compared to control DM sample, we found tendencies (p > 0.08) that correlate with our histological observations. Expression levels of adipogenic markers are presented in Figure 6. Results show average fold changes in gene expression level relative to the control cells cultured in DM. Compared to undifferentiated cells in GM, the adipogenic markers PPARγ and LPL were found to be upregulated in differentiated cells (p < 0.05). In all treatment groups, PPARγ expression was higher than in DM. Glucose treated cells showed the highest upregulation, whereas there was only a minimal increase in the insulin group. Cells, maintained in high-glucose media, showed a statistically significant increase of PPARγ compared to cells in high insulin media (Figure 6(a)). Moreover, insulin appears to reduce the stimulatory effect of glucose on the PPARγ expression. Similarly, LPL expression increased by fourfold glucose treatment, whereas insulin leads to a reduced expression level (Figure 6(b)). Mannitol control for 4G had the highest effect on the upregulation of PPARγ and LPL, with insulin again showing to reduce this effect. Compared to sole insulin treatment, the combination of insulin and mannitol effected a significantly higher LPL expression.

### Glucose and lactate metabolism

**Figure 7.**
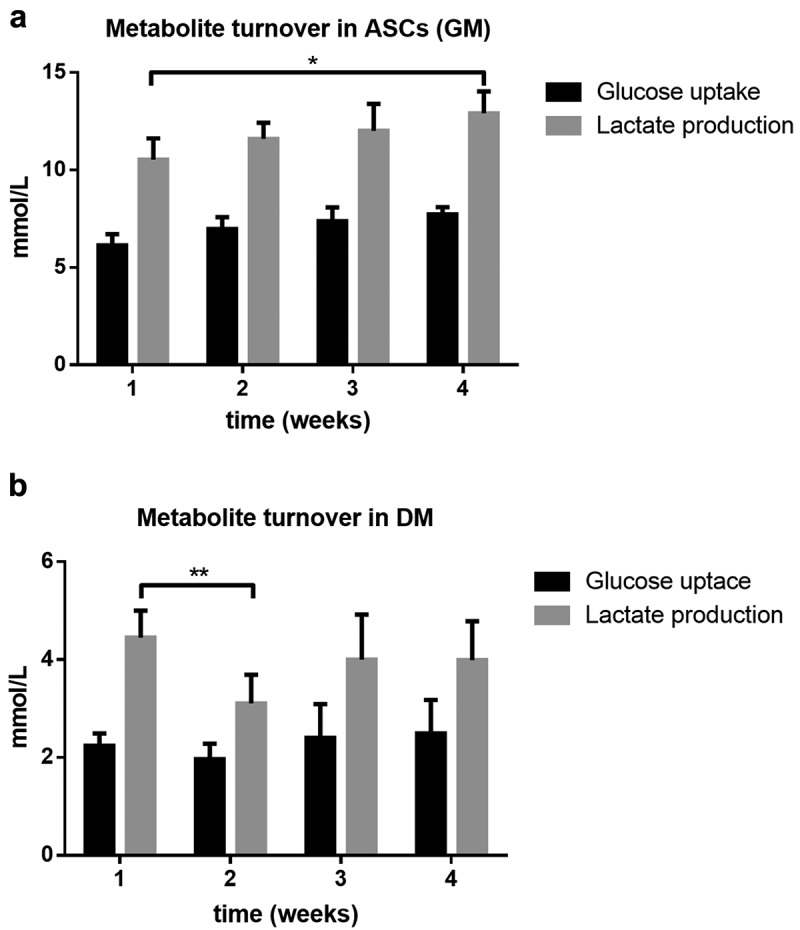
Metabolite turnover, analysed in conditioned media over the period of 4 weeks. Glucose uptake and lactate production in (a) human adipose-derived stem cells (hASC) cultured in growth media (GM) and (b) standard differentiation media (DM). Values are expressed as mean ± SEM, students t-test was used for statistical comparison between groups; *p < 0.05, **p < 0.01.

To analyse the glucose and lactate metabolism, we measured the metabolic parameters in the conditioned media. ASC cultured in GM for 4 weeks showed a steady increase in glucose uptake from 6.13 ± 0.58 mmol/L to 7.73 ± 0.37 mmol/L (Figure 7(a)). Upon differentiation, the average glucose consumption of cells in the different treatment groups appeared to remain constant. Neither higher concentrations of insulin nor glucose exhibited a significant effect on the glucose metabolism. Exemplary for the differentiation group, metabolite turnover is shown in DM (Figure 7(b)). Similarl to the glucose consumption, lactate production in GM increased throughout culture (10.53 ± 1.09 to 12.91 ± 1.12, p < 0.05; Figure 7(a)). After differentiation was induced, a decrease in lactate production followed with the lowest values in the second week postinduction (p < 0.01; Figure 7(b)). In the third and fourth week lactate production increased again.

## Discussion

In the present study, we investigated the outcome of high glucose and insulin application for fat cell augmentation *in vitro*, as published protocols for adipogenic differentiation vary among laboratories. Since mature adipocytes represent both numerically and functionally the principal element of fat tissue [14], we intended to create an *in vitro* monolayer model for the successive approximation to the physiological context of human adipose tissue. Unlike previous studies, we were specifically interested in adipogenesis stimulating processes not in the differentiation induction period but in the late phase of ASC differentiation. In order to evaluate the degree of differentiation and lipid accumulation, we quantified Oil-Red-O stained area and used qRT-PCR to assess whether morphological changes in terminally differentiated cells were still regulated by molecular processes.

Isolated cells were confirmed as mesenchymal stem cells by the typical surface marker expression. As the SVF of adipose tissue is composed of a heterogeneous cell population, variable expression of hematopoietic and endothelial surface markers is commonly found at early passages [15]. Especially, literature differ respectively the expression of CD34. Although the International Society for Cellular Therapy (ISCT) declared CD34 as a negative marker in 2006 [10], in recent years, its expression has been discussed controversially [16–18]. Based on the great variability in data regarding the positivity of CD34, the ISCT re-evaluated the minimal criteria for stromal cells and admit a tissue, harvest and culture dependent expression of CD34, with a consistent downregulation according to the time of cultivation [19].

In the last years, numerous *in vivo* studies have elucidated the role of hyperinsulinemia in promoting adipogenesis, being associated with obesity and insulin resistance, resulting in severe metabolic dysfunctionality [20]. While insulin is known to stimulate adipocyte differentiation dose-dependently at the early stage of differentiation [21], its role in the late phase of differentiation is yet not completely understood. In this study, the capacity to accumulate intracellular lipids was assessed in the presence or absence of glucose and insulin 28 days after differentiation was induced. High insulin treatment of adipocytes was found to be associated with a significant reduction of intracytoplasmic lipid accumulation and a formation of multiple small vacuoles with only a few unilocular large ones, suggesting a more immature stage of conversion. There is a lack of data for human cell lines; however, results are consistent with previous data obtained in 3T3-L1 adipocytes, describing long-term exposure to insulin eliciting a more immature phenotype with fewer unilocular lipid droplets [22]. Though we found these phenotypical observations accompanied by only marginal transcriptional changes of adipocyte-specific genes. Insulin-impaired lipid accumulation was associated with slight changes in the expression of LPL and PPARγ compared to cells cultured in DM. Nevertheless, it is not possible to exclude posttranscriptional changes in enzyme activity and synthesis rate that were not monitored in this study. Raynolds et al. illustrated a transcription-independent role of insulin in the hormonal regulation of LPL activity, including pre-mRNA processing, as well as the transport and stability of mRNA [23,24].

High-glucose treatment increased lipid accumulation in a dose-dependent manner, accompanied by a rise in gene expression of adipogenesis regulating genes. PPARγ which is regarded to be the master regulator of adipogenesis was found to be upregulated in glucose and mannitol treated cells. Compared to high insulin media, fourfold glucose concentration significantly induced PPARγ expression, with insulin supplementation showing to reduce the stimulating effect of glucose.

Aguiari et al. described a significant increase in Oil-Red-O stained area and a transcriptional upregulation of PPARγ and LPL in hASC that were differentiated for 14 days in high-glucose media (25 mM glucose) compared to cells sustained in low glucose media (5.5 mM glucose) [25]. In vitro studies with 3T3-L1 preadipocytes demonstrated that PPARγ, indeed, is essential for the adipogenic differentiation process; however, it is of lesser importance for the maintenance of the differentiated state [26]. Nevertheless, we still found glucose and insulin having an influence on gene expression in late adipogenic differentiation.

To assess whether the observed effects of high-glucose treatment were attributable to its osmotic pressure, cells were incubated in equimolar mannitol media. Although osmotic stress mediated by mannitol did not enhance intracytoplasmic lipid accumulation, it markedly increased expression levels of adipogenic genes. The above-described results for mannitol allow to assume that gene expression and morphological characteristics are not necessarily correlated. As far as we know, to date, no study has described that osmotic pressure per se has an adipogenic potential, triggering the expression of typical genes. While the hormonal regulation of adipogenesis has been widely studied, present results may open the search for currently unknown signal transduction pathways regulating glucose-associated processes with osmotic stress as a mediator. To investigate this further, we analysed glucose and lactate levels of conditioned media over a period of 4 weeks. Lactate, as a product of glucose metabolism in fat cells, has been mentioned sporadically; however, early in vitro studies with human abdominal adipose tissue demonstrated that about 73% of glucose uptake are released as lactate and 11% as CO_2_, while only 16% are utilized for lipid storage [27]. Whereas in this study, during differentiation lactate production of about 4 mmol/L and glucose uptake of about 2 mmol/L were balanced, glucose uptake of ASC in non-adipogenic GM was higher than lactate production (glucose/lactate ratio of 1/1.67). However, in the second week after differentiation, the lowest lactate levels were found with a glucose/lactate ratio of 1/1.58. Although not only glucose but also lactate metabolism is known to be regulated by glucose and insulin concentrations [28], we observed no differences between treatment groups. Interestingly, cells with higher lipid droplet formation did not show higher glucose consumption. Even though glucose-treated cells showed a statistically significant increase in lipid accumulation compared to cells in high insulin media, glucose uptake remained at the same level. In contrast, Crandall et al. found both high-glucose media and insulin to increase basal lactate production in rat adipocytes [29]. Furthermore, they illustrated that large fat cells convert significantly more of the utilized glucose to lactate than smaller ones. Interestingly, they found higher lactate release correlated to a reduced glucose conversion to triglycerides and CO_2_. This flexibility of lactate production in relation to other products of glucose metabolism indicates that storage of triglyceride may not only be achieved by a rise in glucose uptake but also by a modified contribution of products released from glucose metabolism.

## Conclusions

To date, there is no appropriate method applying an extracorporal volume augmentation of prior-harvested autologous fat tissue for the reconstruction of soft tissue defects. In this concern, the subcutaneous fat tissue depot with its accessibility and abundant stem cell reservoir provides a unique source for autologous tissue grafting. By establishing a protocol for the late phase of adipogenesis, our data support the notion that glucose enhances lipid accumulation and gene expression of adipogenic markers, whereas the role of insulin, showing opposed effects, has not been described before. We were able to show that glucose and insulin which were thought to act synergistically have different effects on late adipogenic differentiation; however, there is still a need for clarification. Unexpectedly, we demonstrated a novel aspect of mannitol that equally showed to increase expression levels of typical markers.

## Data Availability

The authors confirm that the data supporting the findings of this study are available within the article.
